# Endométriose cervicale: à propos d'un cas

**DOI:** 10.11604/pamj.2020.35.82.20662

**Published:** 2020-03-19

**Authors:** Saad Benali, Jaouad Kouach

**Affiliations:** 1 Service Gynécologie Obstétrique Hôpital Militaire d'Instruction Mohamed V Rabat, Rabat, Maroc; 2 Faculté de Médecine et de Pharmacie de Rabat, Université Mohamed V Rabat, Rabat, Maroc

**Keywords:** Endométriose, col utérin, lésion cervicale, Endometriosis, uterine cervix, lesion of the uterine cervix

## Abstract

This study reports the case of a 46-year old multiparous patient with no particular past medical history, who had given birth vaginally twice, presenting with abnormal isolated inter-menstrual bleeding with well-defined bluish cyst at the level of the anterior labia of the cervix, measuring 4mm in diameter on speculum examination. Pelvic ultrasound showed adenomyosis. A biopsy of the lesion was performed, which showed dark brown chocolate colour liquid. Histological study objectified endometrium embedded under the epithelium, suggesting endometriosis of the uterine cervix. Endometriosis of the uterine cervix can involve the endocervix and the ectocervix. Primary endometriosis of the uterine cervix can develop in the cervix while secondary endometriosis can result from neighboring endometriosis. Trauma is the main cause of these lesions. The de-epithelialization caused by trauma would allow for endometrial transplantation during menstruation. Endometriosis of the uterine cervix is usually asymptomatic. It is usually discovered during a careful systematic examination of the uterine cervix. Rarely, it manifests as minimal, spontaneous metrorrhagias in the premenstrual period. Sometimes, metrorrhagias occur during menstruations and are persistent or are caused by sexual intercourses. Isolated endometriosis of the uterine cervix never causes pain. The treatment of this benign condition is based on the destruction or removal of the endometriosis foci. Diathermocoagulation is the most used procedure.

## Image en médecine

Patiente âgée de 46 ans, sans antécédent pathologique, multipare, ayant accouché à deux reprises par voie basse, consulte pour un saignement inter-menstruel anormal isolé avec à l'examen au spéculum, présence d'une lésion kystique bleuâtre au niveau de la lèvre antérieure du col, mesurant 4mm de diamètre et bien limitée. L'échographie pelvienne trouve un utérus d'aspect adénomyosique. Une biopsie de la lésion a été réalisée et a donné issue à un liquide noirâtre brun chocolat avec à l'étude histologique, un aspect typique d'endomètre enchâssé sous l'épithélium, faisant évoquer une endométriose cervicale. L'endométriose cervicale peut intéresser l'endocol ou l'exocol, elle peut être primaire née sur le col ou secondaire, elle est alors la propagation d'une endométriose de voisinage. Le rôle des traumatismes semble primordial dans la genèse de ces lésions. La désépithélialisation causée par le traumatisme offrirait le lit de la greffe endométriale au cours de la menstruation. L'endométriose cervicale est généralement asymptomatique. Elle est découverte à l'occasion d'un examen systématique minutieux du col. Rarement, elle se manifeste par des métrorragies minimes, spontanées en période prémenstruelle. Parfois, ces métrorragies suivent les règles, leur imprégnant un caractère traînant ou sont provoquées par des rapports sexuels. L'endométriose isolée du col utérin n'est jamais douloureuse. Le traitement de cette affection bénigne consiste en la destruction ou l'exérèse des foyers endométriosiques. La diathermocoagulation est le procédé le plus utilisé.

**Figure 1:**
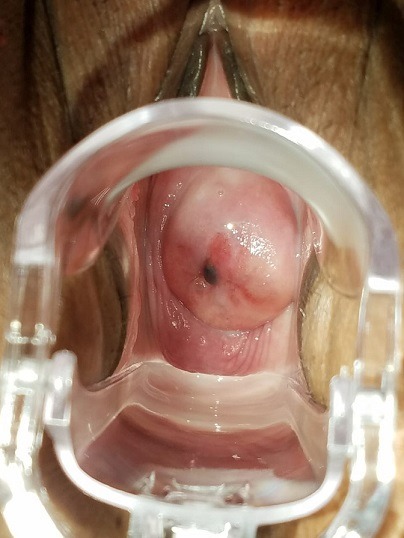
Aspect macroscopique de l'endométriose cervicale à l'examen au speculum

